# Recent Developments in Azomethine Ylide-Initiated Double Cycloadditions

**DOI:** 10.3390/molecules30194019

**Published:** 2025-10-08

**Authors:** Tieli Zhou, Xiaofeng Zhang, Yan Jan Sheng, Desheng Zhan, Wei Zhang

**Affiliations:** 1 College of Food Science and Engineering, Changchun University, Changchun 130022, China; zhoutieli00@sina.com; 2 Center for Green Chemistry, Department of Chemistry, University of Massachusetts Boston, 100 Morrissey Boulevard, Boston, MA 02125, USA; 3 Department of Biochemistry, Brandeis University, Waltham, MA 02453, USA; yansheng@brandeis.edu; 4 College of Chemistry, Changchun Normal University, Changchun 130031, China; zhandesheng@ccsfu.edu.cn

**Keywords:** azomethine ylide, double cycloadditions, pyrrolidine, dipolarophiles, decarboxylation, amino acid

## Abstract

Azomethine ylides (AMYs) have a nitrogen–carbon double bond and an electron lone pair on the nitrogen atom. They are essential 1,3-dipoles for [3+2] cycloadditions in the synthesis of pyrrolidine-containing heterocycles. Significant progress in 1,3-diplolar cycloadditions has been made in the construction of novel heterocyclic scaffolds, with efforts to broaden substrate scope, enhance stereoselectivity, and integrate green chemistry principles. This article summarizes double cycloadditions of AMYs derived from amino esters and amino acids for the synthesis of novel polyheterocycles. The design of double cycloadditions through the pot, atom, and step economic (PASE) method to increase the reaction efficiency is discussed. The examples presented in this paper may be applied to the synthesis of biologically active molecules.

## 1. Introduction

**Figure 1:**
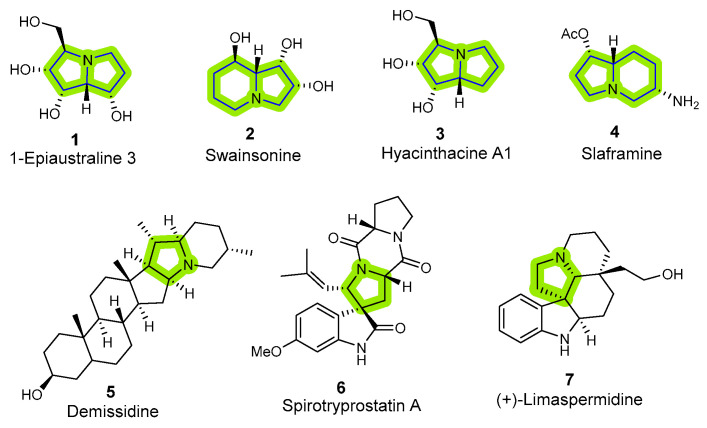
Representative pyrrolidine-containing natural products.

**Figure 2:**
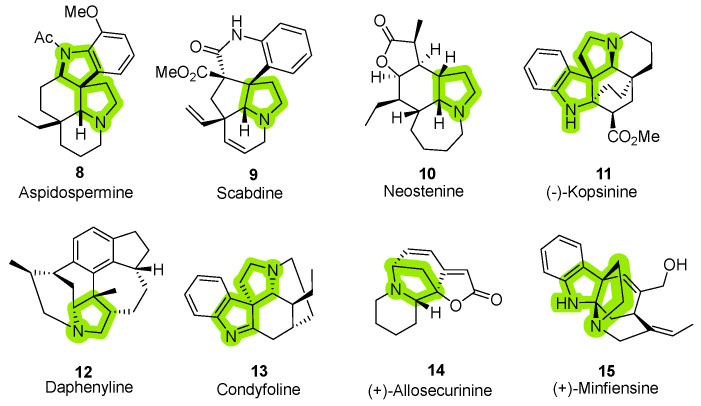
AMY-based synthesis of natural products.

Azomethine ylides (AMYs) are 1,3-dipoles that have a unique nitrogen–carbon double bond and a lone pair of electrons on the nitrogen atom [1,2,3]. They are versatile synthons for [3+2] cycloaddition with dipolarophiles in making pyrrolidine-containing heterocycles [4,5,6,7]. Given the widespread occurrence of pyrrolidine-containing natural products and bioactive compounds **1–7**, as exemplified in Figure 1, significant effort has been dedicated to the development of new methods for the synthesis of pyrrolidine-based heterocyclic systems with broad substrate scope and high stereo- and regioselectivities [8,9,10,11]. These methods have been applied to the synthesis of some natural products and biologically active polyheterocyclic compounds, such as **8–15**, as shown in Figure 2 [12]. Chiral catalyst-promoted asymmetric [3+2] cycloadditions have also been developed to make enantiomerically pure compounds and drug molecules [12,13,14,15,16,17,18,19,20]. Recently, green principles have been applied to increase synthetic efficiency and reduce environmental impact, especially for large-scale synthesis [21,22].

**Scheme 1:**
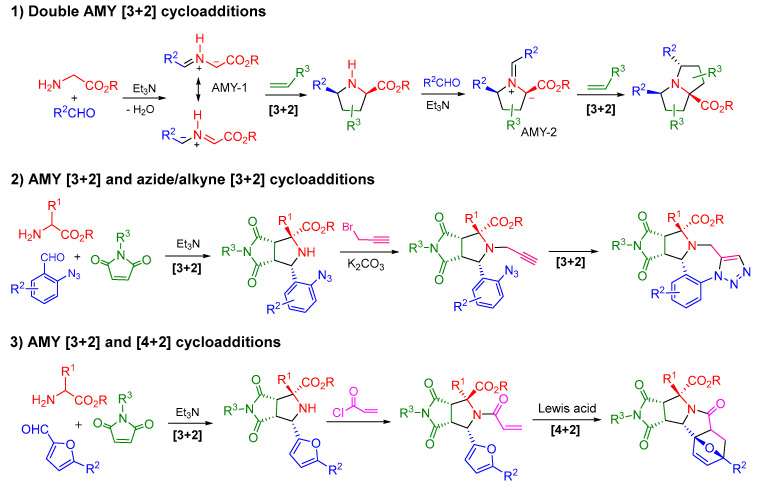
Examples of three kinds of [3+2]-initiated double cycloadditions.

There are a number of reviews on AMYs derived from amino acids/esters [9,11]. They can be generated in situ by various methods, such thermal or photochemical decomposition of iminium salts, condensation of aldehydes with amines, and metal-catalyzed reactions [9]. The resulting AMYs undergo 1,3-dipolar cycloadditions with dipolarophiles, such as electron-deficient alkenes and alkynes. Presented in this paper is AMY-initiated [3+2] cycloaddition, followed by a second cycloaddition of [3+2] or a [4+2] cycloaddition (Scheme 1). Two sequential cycloadditions could be conducted in one pot or as stepwise reactions. These one-pot cycloadditions, without isolation of the intermediates, enhance the atom economy by reducing the number of operational steps, minimizing solvent usage, and reducing the workup and purification steps, increasing time and resource efficiency [23,24,25,26,27,28,29]. This approach has been successfully applied to the synthesis of natural products and bioactive polyheterocyclic systems for medicinal chemistry and agrochemical applications [12].

## 2. Double 1,3-Dipolar Cycloadditions

**Scheme 2:**
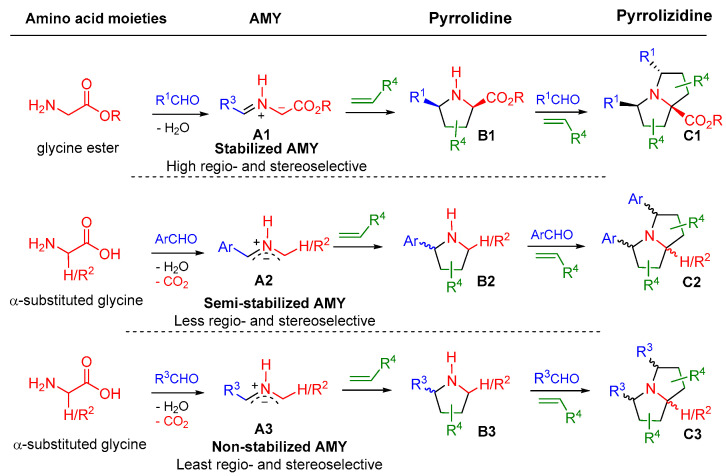
Three AMYs for double [3+2] cycloadditions.

Presented in this section are AMYs for double 1,3-dipolar cycloadditions. There are three types of AMYs generated from the condensation of aldehydes with amino acids/esters through dehydration or decarboxylation processes (Scheme 2) [9]. (1) Stabilized AMYs **A1** derived from amino esters have high regio- and stereoselectivity in 1,3-dipolar cycloadditions due to the electron-withdrawing group (EWG). But only glycine esters with two α-hydrogens can be used for the second cycloaddition of **B1** for making pyrrolizidines **C1**. (2) Semi-stabilized AMYs **A2** generated from the reaction of α-2H/alkyl amino acids with aromatic aldehydes after decarboxylation give rise to products **C2** with reduced stereoselectivity. (3) Non-stabilized AMYs **A3** generated from the reaction of α-2H/alkyl amino acids with aliphatic aldehydes give rise to products with more stereoisomers, **C3**. It was found that pyrrolidines **B2** and **B3** generated in situ from the decarboxylative 1,3-dipolar cycloaddition of semi- or non-stabilized AMYs **A2** and **A3** would undergo second 1,3-dipolar cycloaddition via α-C-H functionalization [30,31,32,33,34]. 

### 2.1. Double [3+2] Cycloadditions with Stabilized AMYs

Stabilized AMYs are in conjugation with electron-withdrawing groups, such as carbonyl or ester groups on the adjacent carbon atom [17,35]. They have high regio- and stereoselectivity in the 1,3-dipolar cycloaddition reactions. But only glycine esters, which have two α-hydrogens, can be used for double [3+2] cycloaddition reactions [9,11]. This limits the scope of using stabilized AMYs for double [3+2] cycloadditions.

#### 2.1.1. Intermolecular Double [3+2] Cycloadditions

**Scheme 3:**
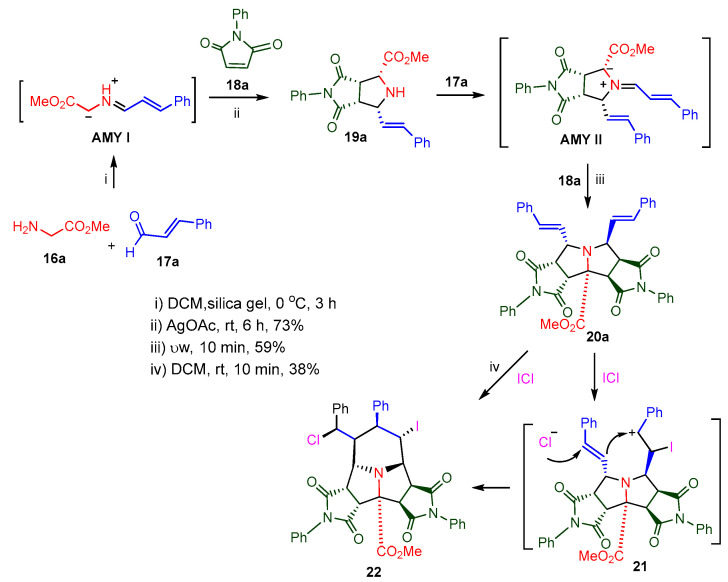
Stereoselective synthesis of pentacyclic pyrrolizidines **22**.

**Scheme 4:**
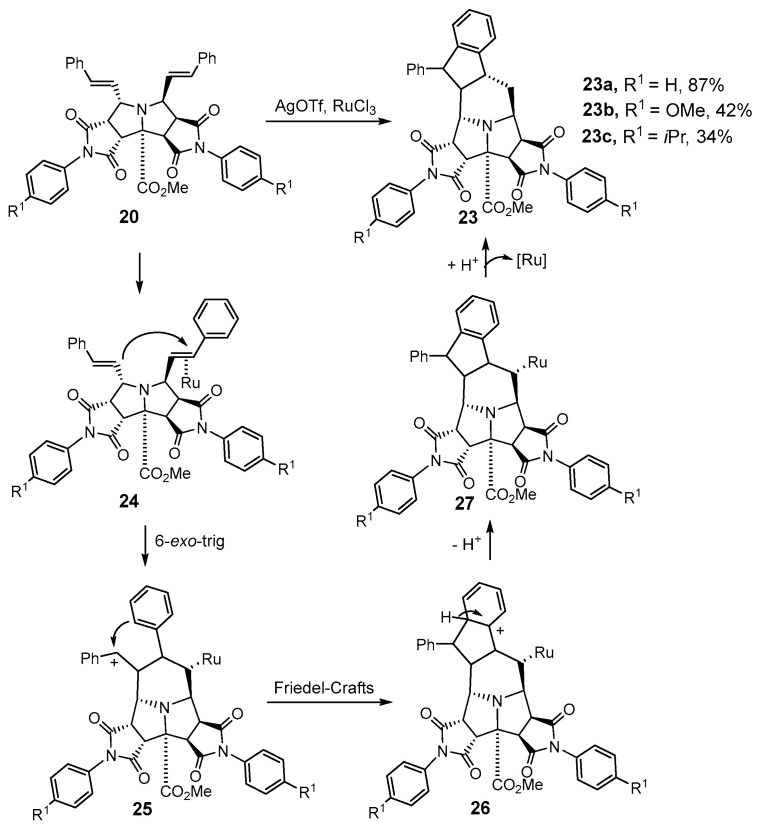
Ru-catalyzed synthesis of heptacyclic pyrrolizidines **23**.

**Scheme 5:**
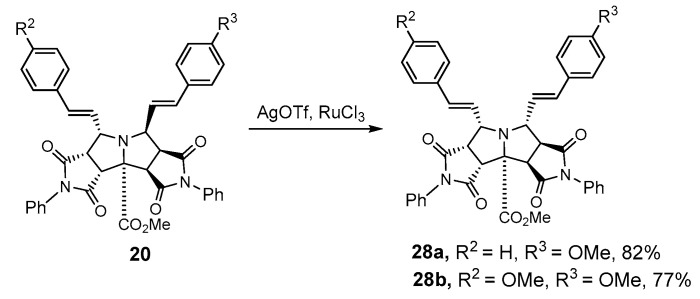
Epimerization of tetracyclic pyrrolizidines **20**.

The Gan group reported the first intermolecular double [3+2] cycloadditions of three components. AMY I, generated from the condensation of glycine ester **16a** and cinnamaldehyde **17a,** reacted with N-phenylmaleimide **18a** to give pyrrolidine **19a,** which then underwent a second [3+2] cycloaddition with the second equivalent of **17a** and **18a** under microwave irradiation to afford pyrrolizidine **20a** (Scheme 3) [36]. The addition of ICl could further convert pyrrolizidine **20a** into highly stereoselective cycl[3.2.2]azine **22** via 6-exo-trig cyclization of **21**. The stereochemistry of **22** was determined by single-crystal X-ray analysis. In the presence of RuCl_3_/AgOTf, pyrrolizidines **20** bearing styrenyl substituents at the 3,5-positions underwent 6-exo-trig cyclization followed by an intramolecular Friedel–Crafts reaction to form decahydrocycl[3.2.2]azines **23a–c** in 34–82% yields. The stereochemistry of **23** was determined by NOESY analysis. The proposed mechanism suggests that the coordination of a Ru catalyst with a less sterically hindered styrenyl double bond gives intermediates **24** (Scheme 4) [37]. The Ru catalyst is activated by AgOTf to form RuCl_2_(OTf) and AgCl. The 6-exo-trig cyclization of **24** *cis* to the ester group gives intermediates **25,** in which a benzyl cation is generated for the Friedel–Crafts reaction to afford **26**. Finally, rearomatization and hydrolysis of **26** give products **23**. When the OMe group was present as R^2^ and/or R^3^ on the styrenyl groups, it was found that **20** could be epimerized to **28** at the pyrrolizidine carbons connected to the styrenyl group (Scheme 5) [37].

**Scheme 6:**
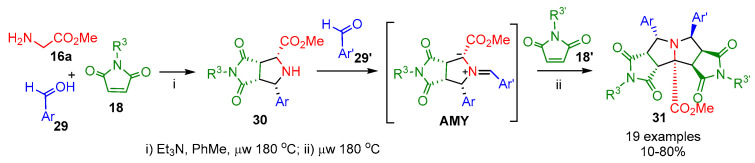
One-pot synthesis of tetracyclic pyrrolizidines **31**.

**Scheme 7:**
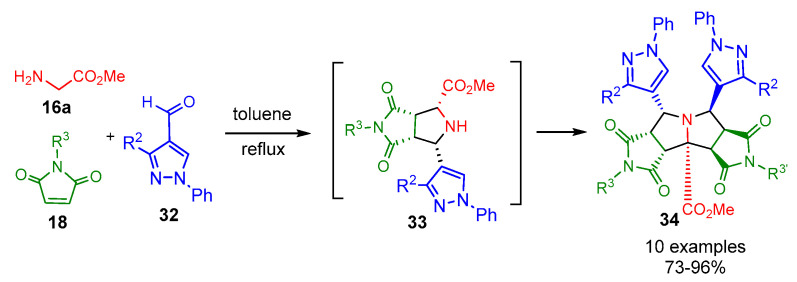
Cascade synthesis of pyrazolylpyrrolizines **34**.

Zhang’s group developed microwave-assisted one-pot double [3+2] cycloadditions of glycine ester **16a** with aromatic aldehydes **29** and maleimides **18** for the first cycloaddition to make intermediates **30,** followed by a second cycloaddition with aromatic aldehydes **29′** and maleimides **18′** to afford tetracyclic pyrrolizidines **31** in 10–80% yields. The product structure was determined by single-crystal X-ray analysis (Scheme 6) [38]. Quiroga’s group also accomplished diastereoselective synthesis of pyrazolylpyrrolizines **34** through double cycloadditions of glycine ester **16a** with two equivalents each of formylpyrazoles **32** and maleimides **18** under conventional heating (Scheme 7) [39].

**Scheme 8:**
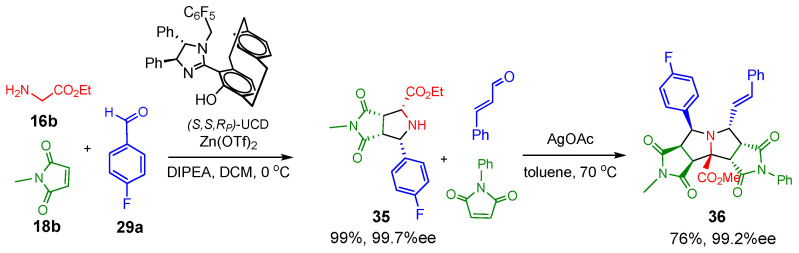
Enantioselective synthesis of pyrrolizidines **36**.

The Guiry group employed a series of chiral UCD Imphanol ligands possessing central and planar chirality in Zn-catalyzed asymmetric [3+2] cycloadditions [40]. Among them, compound **35** was made in a 92% yield with excellent diastereo- and enantioselectivities (*endo/exo* > 99/1 and 99.7% *ee*) using Zn(OTf)_2_ and (*S,S*,RP)-UCD-Imphanol. The second cycloaddition of pyrrolidines **35** gave tetracyclic pyrrolizidine **36** in a 76% yield with *endo/exo* > 92/8 and 99.2% ee. During the second cycloaddition, no stereochemistry erosion was observed (Scheme 8).

**Scheme 9:**
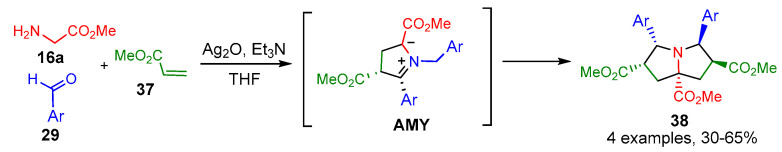
Intermolecular double [3+2] cycloadditions with methyl acrylate.

**Scheme 10:**
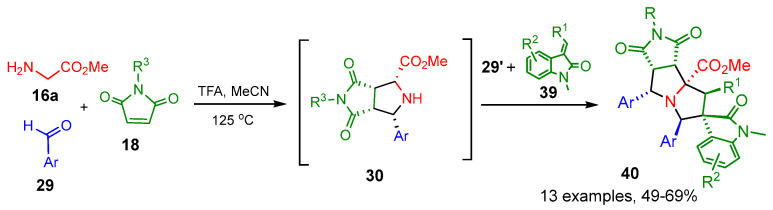
Diastereoselective synthesis of spirooxindole pyrrolizidines **40**.

**Scheme 11:**
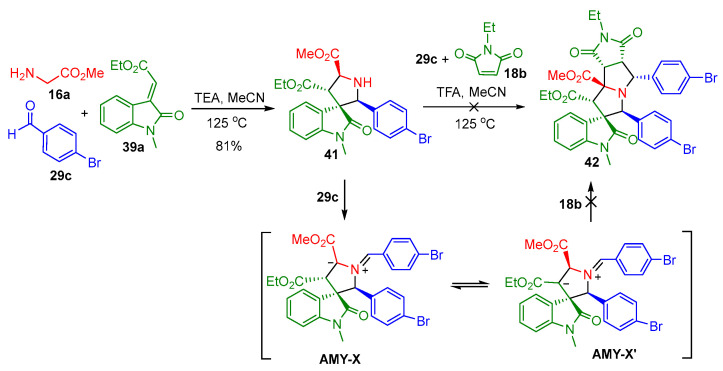
Attempted reactions for making compound **42**.

Instead of using maleimides **18** as dipolarophiles, the Miftakhov lab employed methyl acrylate **37** for Ag-catalyzed double [3+2] cycloadditions to afford pyrrolizidine alkaloids **38** in 30–65% yields (Scheme 9) [41]. The structure confirmation was accomplished via NOE and X-ray single-crystal analyses. Zhang’s group employed different aldehydes, **29** and **29′,** as well as maleimides **18** and olefinic oxindoles **39** as different dipolarophiles for two [3+2] cycloadditions in the diastereoselective synthesis of spirooxindole pyrrolizidines **40** in 49–69% yields (Scheme 10) [42]. When olefinic oxindole **39a** was used for the first cycloaddition, it was found that adducts **41** failed to react with **29c** and **18b** for the second cycloaddition. The proton in the β-ester group of AMY-X is probably less acidic than that in AMY-X’, which prevents the second cycloaddition from forming product **42** (Scheme 11) [42].

#### 2.1.2. Intramolecular Double [3+2] Cycloadditions

**Scheme 12:**
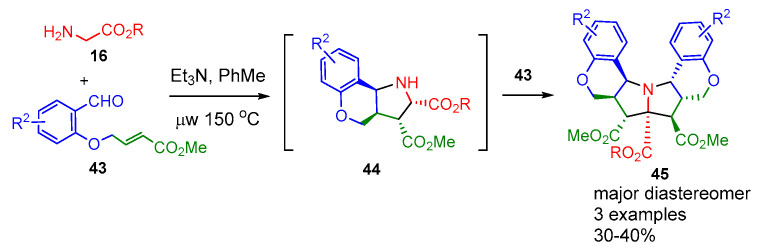
One-pot synthesis of hexacyclic pyrrolizidines **45**.

The first intramolecular double [3+2] cycloaddition of glycine ester was reported by Zhang’s group in 2005. In this case, the one-pot reaction of glycine esters **16** with an excess amount of O-allyl salicylaldehydes **43** afforded a novel hexacyclic ring system **45,** which had four new rings and seven stereocenters (Scheme 12) [43]. The first cycloaddition generated tricyclic proline intermediates **44,** which then underwent a second cycloaddition to afford compounds **45** diastereoselectively. The major stereoisomers were isolated in 30–40% yields, and the stereochemistry of the product was confirmed by X-ray crystal structure analysis. If the major diastereomers have perfluoro C_8_F_17_(CH_2_)_3_ as the R group, they can simply be purified by fluorous solid-phase extraction (F-SPE) [44,45].

#### 2.1.3. Intermolecular and Intramolecular [3+2] Cycloadditions

**Scheme 13:**
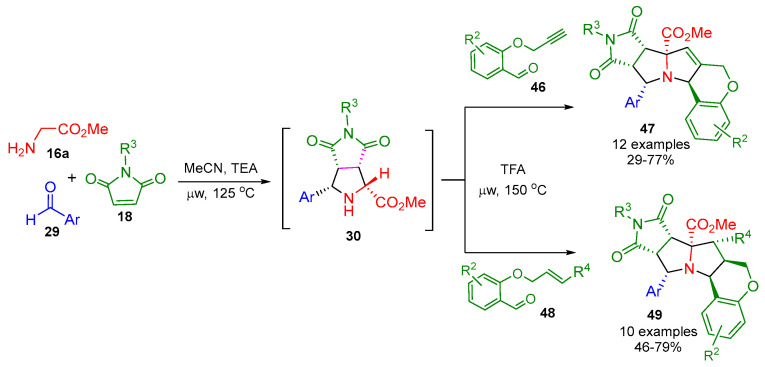
One-pot synthesis of pentacyclic pyrrolizidines **47** and **49**.

The combination of inter- and intramolecular [3+2] cycloadditions could lead to the formation of novel and complex scaffolds. Zhang’s group utilized benzaldehydes **29**, glycine ester **16a**, and N-ethylmaleimides **18** for intermolecular cycloaddition to make intermediate **30** under microwave heating. Without isolation, the reaction mixture was reacted with dipolarophiles **46** or **48** under TFA catalysis for the intramolecular [3+2] cycloaddition (Scheme 13) [46]. The reaction of alkynes **46** generated **47** with three new rings, six new bonds, and five stereocenters in 29–77% yields with 8:1 to 13:1 dr. The reaction of terminal alkenes **48a,b** afforded products **49a,b** with six stereocenters in up to a 57% yield and > 5:1 *dr*. The reactions of alkenes **48c–j** with an R^4^ group gave products **49c–j**, bearing seven stereocenters in 46–79% yields with 5:1 to 8:1 *dr*.

### 2.2. Decarboxylative Double [3+2] Cycloadditions

**Scheme 14:**
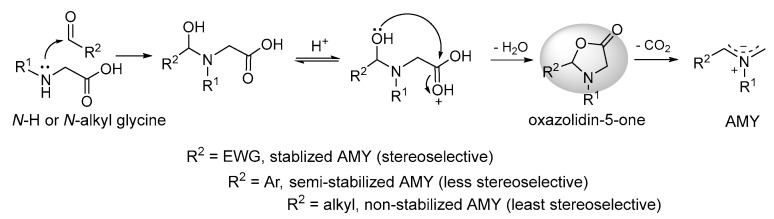
Formation of AMYs via decarboxylation of oxazolidin-5-ones.

Stabilized AMYs derived from amino esters have been well utilized in [3+2] cycloadditions. AMYs derived from amino acids were first reported in 1980s, but they have not been well explored in [3+2] cycloadditions because the reactions are more complicated [47,48,49,50,51,52]. In 2019, Zhang’s group reported the double [3+2] cycloadditions of amino acids involving the key intermediate of oxazolidin-5-ones (Scheme 14) [9]. Compared to using glycine esters to generate AMYs for double cycloadditions, a range of α-amino acids could be used in cycloadditions since the CO_2_ is released to generate a reactive site for the second cycloaddition. However, if R^2^ is not an EWG, AMYs derived from α-amino acids may be semi-stabilized or non-stabilized, producing less stereoselective cycloaddition products. It is important to understand the mechanism of amino acid-based double cycloadditions.

#### 2.2.1. Double [3+2] Cycloadditions with Maleimides

Zhang’s group reported the first case of amino acid-based decarboxylative double [3+2] cycloaddition. It was a A+2B+2C-type five-component reaction (5-CR), which uses one equivalent of α-amino acids **50** and two equivalents each of aromatic aldehydes **29** and maleimides **18** for making tetracyclic pyrrolizidines **51**. Under optimized conditions, the reaction of glycine produced **51a** in a 91% yield with >9:1. In addition to glycine, other amino acids such as alanine, butyrine, norvaline, serine, leucine and aspartic acid could be used for the 5-CRs (Table 1) [53,54]. AcOH was added as a catalyst to overcome the hindrance effects of R^1^ to make **51b-f** in up to an 88% yield with dr > 8:1 [53]. The reaction of aspartic acid (R^1^ = CH_2_CO_2_H) afforded compound **51g** in a 66% yield with ~4:1 *dr* [54]. However, some α-amino acids, such as valine and phenylglycine, did not give rise to double cycloaddition products **51** but only the first [3+2] adducts **52**. The double cycloaddition reactions were also conducted as one-pot and two-step processes through a reaction of one equivalent each of **18**, **29a,** and **50** for the first cycloaddition, followed by the addition of another equivalent of **29a** and **50** for the second cycloaddition. As shown in the last column of Table 1, the two-step process was only successful for the synthesis of **51a**.

**Scheme 15:**
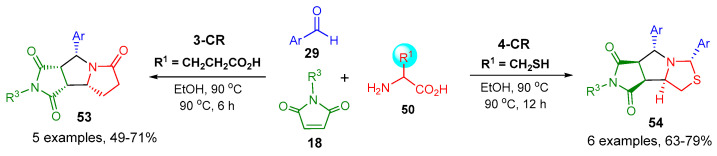
3-CR and 4-CR for tetrahydropyrrolizinones **53** and tetrahydropyrrolothiazoles **54**.

Control reactions were conducted to understand the mechanism of 5-CRs. In addition, 3-CRs of amino acids **50** (glutamic acid or cysteine) with equal amounts of molar aldehydes **29** and maleimides **18** gave tetrahydropyrrolizinones **53** in 49–71% yields. A four-component reaction (4-CR) with two equivalents of aldehydes gave tetrahydropyrrolothiazoles **54** in 63–79% yields, while 5-CRs failed in the synthesis of tetracyclic pyrrolizidines **51** (Scheme 15) [54,55].

**Scheme 16:**
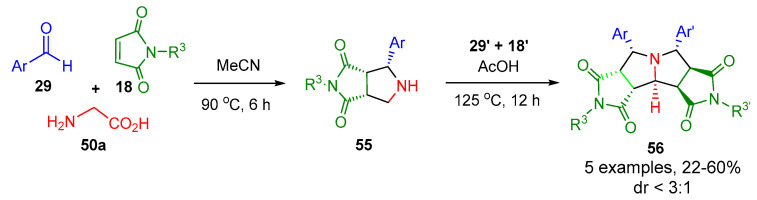
Two-step synthesis of tetracyclic pyrrolizidines **56**.

Stepwise double cycloadditions of glycine **50a** with two different sets of aldehydes (**29** and **29′**) and two different sets of dipolarophiles (**18** and **18′**) were conducted by Zhang’s group. In the reaction process, the first cycloaddition products **55** generated from the reaction of **50a**, **29,** and **18** were isolated and used for the second cycloaddition with **29′** and **18′** (Table 1) [53]. Compounds **56** were isolated in 22–60% yields with 3:1 *dr* (Scheme 16), which is lower than that of the 5-CR for **51a** (91%, >9:1 *dr*) shown in Table 1. Other than glycine, other α-amino acids could not afford double cycloaddition products [53].

**Scheme 17:**
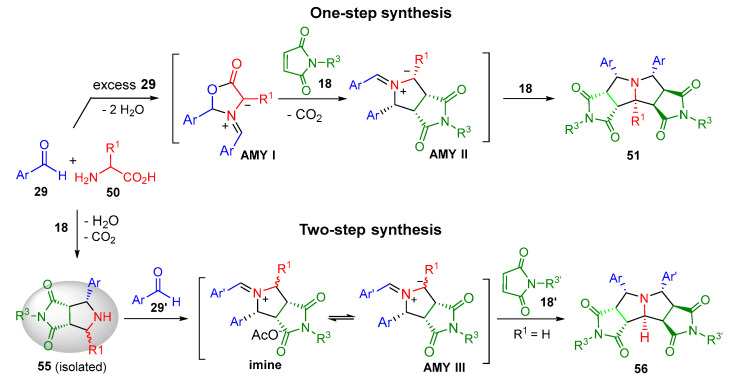
Proposed pathways for single-step vs. two-step double [3+2] cycloadditions.

The proposed mechanisms for one-step and two-step double cycloadditions are shown in Scheme 17. In the single-step reactions, the reaction of acid **50** with 2 equiv. of aldehydes **29** generates stabilized AMY-I with Ar and lactone as conjugate groups to facilitate decarboxylative cycloaddition with **18** to form semi-stabilized AMY-II for a second cycloaddition with another equivalent of **18** to give pyrrolizidines **51**. In the two-step reaction process, pyrrolidines **55** generated from the first cycloaddition of semi-stabilized AMY-III were isolated and used for the formation of another semi-stabilized AMY-IV for a second cycloaddition to give **56** in low yields and with low stereoselectivity (Scheme 16) [53,54]. Only the production of an amino acid with R^1^ = H is possible due to there being less steric hindrance for α-C-H activation [30,31,32,33,34]. Mechanism analysis indicates that two-step double cycloadditions of two semi-stabilized AMYs limits the reaction scope and gives products with low yields and stereoselectivity.

#### 2.2.2. Double [3+2] Cycloadditions with Olefinic Oxindoles

**Scheme 18:**
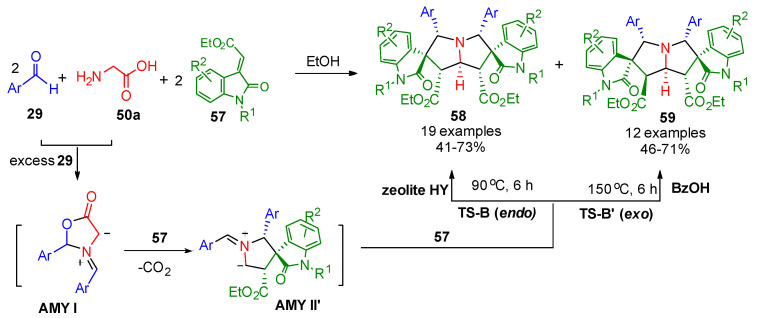
5-CR to make dispirooxindole-pyrrolizidines **58** and **59**.

**Scheme 19:**
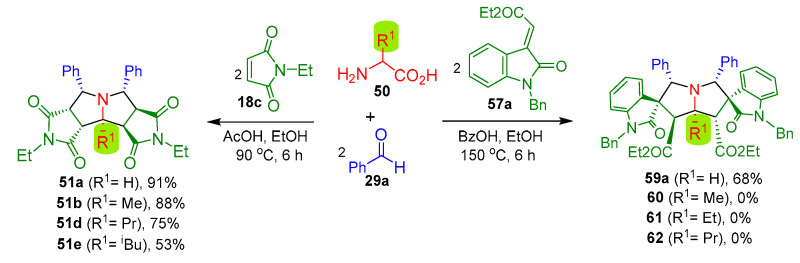
Double cycloadditions with maleimide **18c** vs. olefinic oxindole **57a**.

As part of their effort to develop A+2B+2C-type decarboxylative double [3+2] cycloadditions of amino acids **50**, Zhang and coworkers employed olefinic oxindoles as dipolarophiles in the synthesis of dispirooxindole-pyrrolizidines **58** and **59,** which have a butterfly-shaped skeleton (Scheme 18). In the reaction of glycine **50a**, aldehydes **29**, and olefinic oxindoles **57** under heterogeneous catalysis of zeolite HY, compounds **58** were obtained as the major diastereomers in 41–73% yields. Zeolite HY has a three-dimensional pore structure to regulate stereoselectivity via an endo-transition state to produce products that have a symmetric butterfly shape [56]. Using BzOH as a catalyst and heating at 150 °C, the cycloadditions occurred via an *exo*-transition state to afford **59** as the major diastereomers in 46–71% yields [57]. Compared to reactions using maleimides **18** as dipolarophiles to form **51a–e**, the reactions of olefinic oxindoles **57** only reacted with glycine to afford compound **59a**. The reactions of R^1^-substituted amino acids were unsuccessful in the attempted synthesis of compounds **60–62** due to steric hindrance for the second cycloaddition (Scheme 19).

#### 2.2.3. Double [3+2] Cycloadditions with Meta-Dinitrobenzene Derivatives

**Scheme 20:**
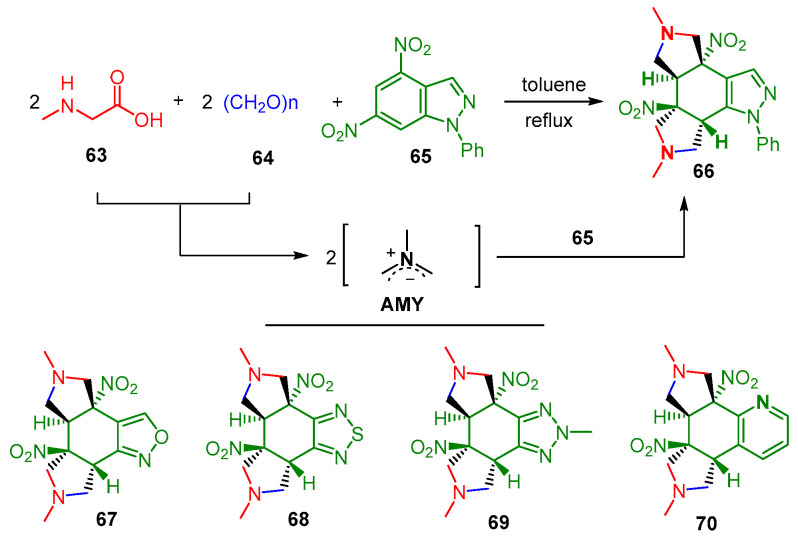
Parallel double decarboxylative [3+2] cycloadditions.

The Starosotnikov group utilized meta-dinitrobenzene derivatives, such as compound **65**, in combination with dual dipolarophiles in double cycloaddition reactions of non-stabilized *N*-methyl AMY derived from the reaction of sarcosine **63** and paraformaldehyde **64**. The reaction process afforded compounds **66–70** in 24–69% yields (Scheme 20) [58]. The AMY has favorable symmetry, which contributes to the stereoselective formation of compounds **66–70**. This is a noteworthy case in the parallel cycloaddition of meta-dinitrobenzene derivatives.

### 2.3. [3+2] Cycloaddition Followed by a Click Reaction

**Scheme 21:**
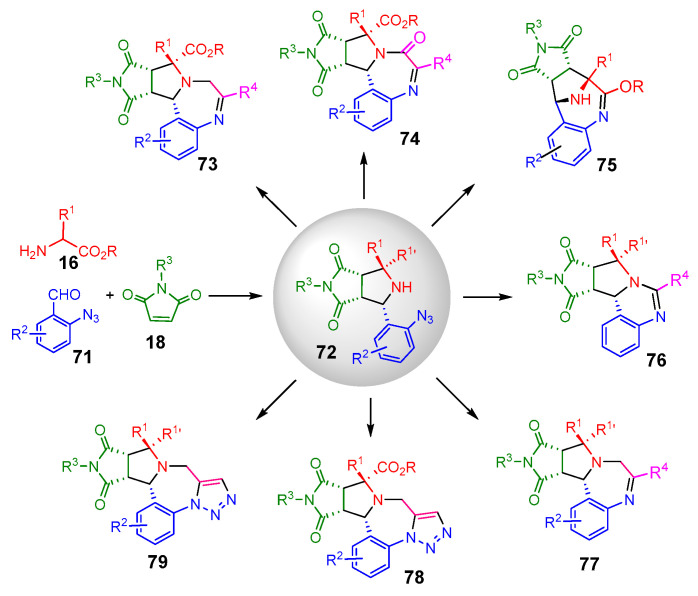
2-Azidobenzaldehyde-based [3+2] cycloadditions for heterocyclic systems.

The combination of two different kinds of cycloaddition reactions is an efficient way to synthesize complex molecular scaffolds [23,24,25,26,27]. It has been applied to the synthesis of biologically relevant molecules [9,12]. Zhang’s group employed [3+2] cycloaddition intermediates for subsequential annulation reactions to generate diverse heterocyclic systems. Among these, notable examples using 2-azidobenzaldehyde-based [3+2] cycloadditions paired with cyclization and cycloadditions for synthesizing polyheterocycles **73–79** are shown in Scheme 21. In this section, we only discuss [3+2] cycloaddition followed by a click reaction in the synthesis of **78** and **79** [59,60].

#### 2.3.1. Stabilized AMY-Based [3+2] Cycloadditions

**Scheme 22:**
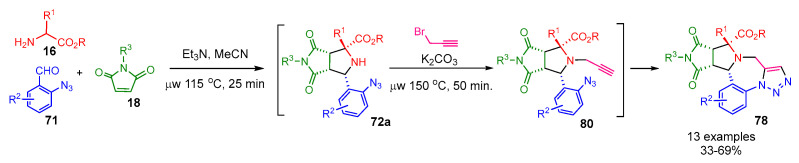
Stabilized AMY-initiated [3+2] cycloaddition and click chemistry.

Cycloaddition of amino esters **16**, maleimides **18**, and 2-azidobenzaldehydes **71** via the formation of stabilized AMYs leads to the formation of intermediates **72a** in good stereoselectivity. Zhang’s group conducted *N*-propargylation of **72a** to form **80**, which were readily converted to triazolobenzodiazepines **78** after the intramolecular click reaction. By optimizing the reaction conditions, the [3+2] cycloaddition, N-propargylation, and click chemistry were achieved through a one-pot reaction process which eliminated the steps of intermediate purification and metal catalysts for the click reaction. A total of 13 analogs of **78** were obtained in 33–69% yields as single diastereomers. The stereochemistry of the products was confirmed by X-ray crystal analysis (Scheme 22) [59].

#### 2.3.2. Semi-Stabilized AMY-Initiated [3+2] Cycloaddition

**Scheme 23:**
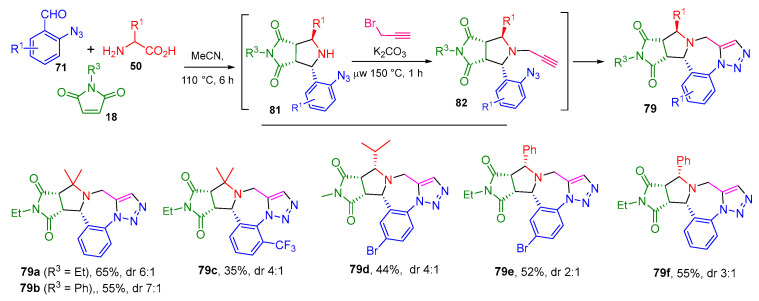
[3+2] Cycloaddition of semi-stabilized AMYs and click reaction.

The NH pyrrolidines **81** generated by the decarboxylative [3+2] cycloaddition of α-amino acids could be combined through the click reaction in the synthesis of triazolobenzodiazepines **79**. As opposed to the cycloadditions shown in Scheme 22, which involve stabilized AMYs, the reaction of α-amino acids involves semi-stabilized AMYs. Ma and coworkers developed a one-pot processes by combining decarboxylative [3+2] cycloaddition, N-propargylation, and the click reaction. Among the amino acids, the reaction of valine, phenylglycine, and aminoisobutyric acid afforded compounds **79a–f** in 35–65% yields, but the diastereoselectivity was reduced from 7:1 to 2:1 due to the semi-stabilized AMYs (Scheme 23) [60].

## 3. [3+2] Cycloaddition and [4+2] Cycloaddition

**Scheme 24:**
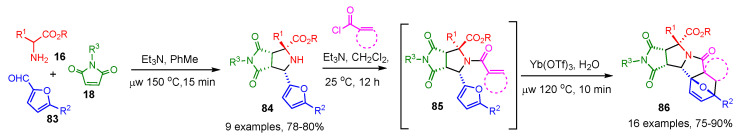
[3+2] Cycloaddition of semi-stabilized AMYs and click reaction.

The [4+2] cycloaddition (Diels–Alder reaction) is the most popular cycloaddition reaction for making six-membered compounds [61,62,63]. The [3+2] adducts could be used for [4+2] cycloaddition in the synthesis of complex structures with diverse functional groups [26,27,64,65,66,67]. Zhang’s group developed sequential [3+2] and [4+2] cycloaddition reactions in the synthesis of highly condensed heterocyclic compounds **86**. The stereoselective adducts **84** generated from the [3+2] cycloaddition of 2-furanylaldehydes **83**, amino esters **16**, and maleimides **18** were N-acylated to give intermediates **85**, which readily underwent intramolecular [4+2] cycloaddition to afford polyheterocyclic products **86** in 75–90% yields (Scheme 24) [68].

## 4. Conclusions

Presented in this paper are double cycloadditions of AMYs derived from amino esters or amino acids for the synthesis of complex polyheterocycles. The pyrrolidine intermediates generated from the initial [3+2] cycloaddition were employed in the subsequent [3+2], click, or [4+2] cycloaddition reactions to demonstrate the versatility of this strategy. Different synthetic designs including multicomponent, one-pot stepwise, and multistep reactions afforded a diverse array of novel heterocyclic architectures, such as tetracyclic, pentacyclic, hexacyclic, and heptacyclic compounds. Notably, the methodology enabled the efficient construction of biologically interesting scaffolds, including pyrrolizidines, spirooxindole- and dispirooxindole-pyrrolizidines, tetrahydropyrrolizinones, tetrahydropyrrolothiazoles, and triazolobenzodiazepines. We believe this synthetic platform is valuable in the generation of structurally complex and diverse polyheterocycles for applications in organic synthesis and medicinal chemistry.

## Figures and Tables

**Table 1:** 5-CR for making tetracyclic pyrrolizidines **51**.

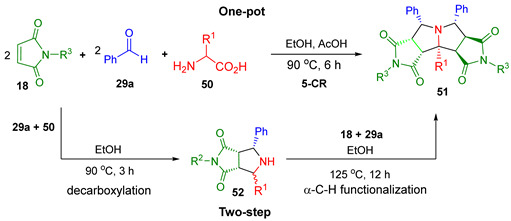					
Amino Acids 50	R^1^	R^2^	Product	One-Pot Yield (dr) ^3^	Two-Step Yield (dr) ^4^
glycine ^1^	H	Et	**51a**	91% (>9:1)	48.5% (3:1)
alanine	Me	Et	**51b**	88% (>8:1)	--
butyrine	Et	Et	**51c**	88% (>8:1)	--
norvaline	Pr	Et	**51d**	75% (>8:1)	--
serine	CH_2_OH	Me	**51e**	83% (>8:1)	--
leucine	^i^Bu	Me	**51f**	53% (>8:1)	--
aspartic acid ^2^	CH_2_CO_2_H	Et	**51g**	66% (~4:1)	--
valine	^i^Pr	Et	**51h**	--	--
phenylglycine	Ph	Et	**51i**	--	--
glutamic acid	CH_2_CH_2_CO_2_H	Et	**51j**	--	--
cysteine	CH_2_SH	Et	**51k**	--	--

^1^ a EtOH, 90 °C, 3 h; ^2^ EtOH, AcOH, 110 °C, 12 h; ^3^ one-pot 5-CR process; ^4^ two-step process.

## Abbreviations

AMY	Azomethine ylide
BzOH	Benzoic acid
EWG	Electron-withdrawing group
NOESY	Nuclear Overhauser effect spectroscopy
PASE	Pot, atom, and step economic
TEA	Triethylamine
TFA	Trifluoroacetic acid
THF	Tetrahydrofuran
UCD-Imphanol	Imidazolinyl-[2.2]paracyclophanol
